# Environmental filtering and dispersal limitation jointly shaped the taxonomic and phylogenetic beta diversity of natural forests in southern China

**DOI:** 10.1002/ece3.7711

**Published:** 2021-05-26

**Authors:** Wei Shi, Yong‐Qiang Wang, Wu‐Sheng Xiang, Xian‐Kun Li, Kun‐Fang Cao

**Affiliations:** ^1^ Guangxi Key Laboratory of Forest Ecology and Conservation College of Forestry Guangxi University Nanning China; ^2^ State Key Laboratory for Conservation and Utilization of Subtropical Agro‐bioresources Guangxi University Nanning China; ^3^ Guangxi Key Laboratory of Plant Conservation and Restoration Ecology in Karst Terrain Guangxi Institute of Botany Chinese Academy of Sciences Guilin China

**Keywords:** community assembly, environmental filtering, null model, phylogenetic β‐diversity, Rao's quadratic entropy, taxonomic β‐diversity

## Abstract

**Aim:**

The mechanisms underlying the maintenance of biodiversity remain to be elucidated. Taxonomic diversity alone remains an unresolved issue, especially in terms of the mechanisms of species co‐existence. We hypothesized that phylogenetic information could help to elucidate the mechanism of community assembly and the services and functions of ecosystems. The aim of this study was to explore the mechanisms driving floral diversity in subtropical forests and evaluate the relative effects of these mechanisms on diversity variation, by combining taxonomic and phylogenetic information.

**Location:**

We examined 35 1‐ha tree stem‐mapped plots across eight national nature reserves in Guangxi Zhuang Autonomous Region, China.

**Taxon:**

Trees.

**Methods:**

We quantified the taxonomic and phylogenetic β‐diversity between each pair of plots using the (abundance‐based) Rao's quadratic entropy and the (incidence‐based) Sørensen dissimilarity indices. Using a null model approach, we compared the observed β‐diversity with the expected diversity at random and calculated the standard effect size of the observed β‐diversity deviation. Furthermore, we used distance‐based redundancy analysis (dbRDA) to partition the variations in taxonomic and phylogenetic observed β‐diversity and β‐deviation into four parts to assess the environmental and spatial effects.

**Results:**

The taxonomic β‐deviation was related to and higher than the phylogenetic β‐deviation (*r* = .74). This indicated that the species turnover between pairwise plots was mainly the turnover of closely related species. Higher taxonomic and phylogenetic β‐deviation were mainly concentrated in the pairwise karst and nonkarst forest plots, indicating that the species in karst forests and nonkarst forests were predominantly from distantly related clades. A large proportions of the variation in taxonomic and phylogenetic β‐deviation were explained by the joint effect of environmental and spatial variables, while the contribution of environmental variables was greater than that of spatial variables, probably owing to the influence of the sampling scale dependence, integrality of sampling size and species pool, and the unique climatic and geomorphic characteristics.

**Main conclusions:**

Our study highlights the importance of phylogeny in biodiversity research. The incorporation of taxonomic and phylogenetic information provides a perspective to explore potential underlying mechanisms that have shaped species assemblages and phylogenetic patterns in biodiversity hotspots.

## Graphical abstract

Our study suggested that the species turnover between pairwise plots was mainly the turnover of closely related species from the same or similar clades, and environmental variables could be expected to be better predictors of taxonomic and phylogenetic β‐diversity than spatial variables in subtropical forests. Furthermore, it revealed a large dissimilarity in the composition of species from distantly related clades in karst communities compared to that of neighboring nonkarst forest communities. Finally, our study emphasized the importance of understanding community assembly mechanisms from the perspective of a combination of taxonomic and phylogenetic β‐diversity.

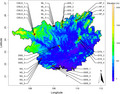

## 1 INTRODUCTION

Understanding diversity patterns and exploring their underlying mechanisms have long been central issues in community ecology (Begon et al., 2006; Chase & Leibold, 2003; Condit et al., 2002). β‐diversity, defined as the spatial changes in community composition, is critical for understanding the influence of environmental and spatial factors on community assembly (Heino & Tolonen, 2017; Leibold & Chase, 2017). Traditional β‐diversity analyses have been generally based on taxonomic data (Legendre & De Cáceres, 2013; Sabatini et al., 2018; Xing & He, 2019). With the rapid development of molecular technology, the phylogenetic β‐diversity analysis has gradually increased. Such analysis allows researchers to verify various hypotheses on biodiversity patterns from a historical perspective, thereby facilitating the understanding of potential evolutionary imprints in community assembly processes (Branco et al., 2020; Cavender‐Bares et al., 2009; Weinstein et al., 2014).

The species compositions of plant communities are resulted from the interaction of ecological and evolutionary processes (Ricklefs, 1987). In fact, species carry genetic information so that taxonomic diversity is strongly dependent on phylogenetic diversity (Penone et al., 2016). Closely related species may have similar traits (Felsenstein, 1985) and thus similar adaptability to a given habitat (Blomberg & Garland, 2002). Therefore, phylogenetic diversity can provide more information about the mechanisms that drive community assembly than taxonomic diversity (Purschke et al., 2013; Swenson et al., 2012; Wang et al., 2019). However, the same mechanism can result in a discordance between taxonomic and phylogenetic β‐diversity. For instance, Oliveira et al. (2013) revealed that closely related species may occupy different habitats. This probably results in a low phylogenetic but high taxonomic diversity between communities.

Previous studies have revealed that community assembly mechanisms can be divided into deterministic processes (e.g., habitat filtering) based on the niche theory (Baldeck et al., 2013; Chase & Leibold, 2003) and stochastic processes (e.g., dispersal limitation) based on the neutral theory (Hubbell, 2001). The roles of deterministic and stochastic processes have been debated for decades, and recently, a consensus was reached that no single mechanism could adequately explain all observed patterns (Adler et al., 2007; Barot, 2004; Gaston & Chown, 2005; Legendre et al., 2009; Qian, Chen, et al., 2013). The joint operation of deterministic and stochastic processes shapes diversity patterns, but the relative contribution of these two processes in different geographic regions and at different scales remains inconclusive (Chu et al., 2019; Myers et al., 2013; Shen et al., 2009; Zhou & Zhang, 2008). The cladistic information associated with evolutionary time‐scale processes is influenced by spatial factors (dispersal limitation caused by geographical isolation) and/or environmental factors (niche stability and conservatism, historical habitat stability, and species‐habitat affinity), and such influences leave direct or indirect effects in diversity patterns (Graham & Fine, 2008; Swenson, 2013; Swenson et al., 2007). These effects may be elucidated by partitioning the contribution of various factors to community assembly.

Most of the current forest diversity studies focus on tropical and temperate forests, while more research attention should be paid to subtropical forests. The subtropical forest in East Asia, which is the largest evergreen broad‐leaved forest worldwide, has been predicted to be one of the biomes with the largest increase in nitrogen deposition in future, and its average net ecosystem productivity (NEP) is higher than that of tropical rainforests and temperate forests in Asia (Galloway et al., 2004; Yu et al., 2014). It hosts unique and rich biodiversity and ensures vital ecosystem services. Owing to the blocking of the westerly wind circulation and the intensification of the Asian monsoon caused by the Qinghai–Tibetan Plateau, the subtropical region of East Asia, located in the vast area of the south of the Qinling Mountains–Huaihe River in China, hosts typical subtropical rainforests. It also covers a biodiversity hotspot (i.e., the mountains of southwest China) and one of the three major karst regions globally (Myers et al., 2000; Wang et al., 2007).

In East Asia, which had several refugia for the survival of plants during glaciations, some of the current flora species were likely migrated to further southern areas such as the Indo‐China Peninsula during the postglacial period (Qian, Swenson, et al., 2013; Wiens & Donoghue, 2004). Given the potential effect of abiotic factors (e.g., environmental filtering, biogeological barriers, and competition) on community structure, the differential evolution of physiological tolerances of phylogenetic clades may promote speciation events and generate regional phylogenetic patterns (Qian, Swenson, et al., 2013). If there is an interaction between phylogenetic relatedness and species colonization, a relationship between phylogenetic and taxonomic β‐diversity (standardized dissimilarity) is expected (Qian, Swenson, et al., 2013). If phylogenetic β‐diversity is higher than taxonomic β‐diversity, this would indicate that the species compositions of the two communities are distantly related. If the phylogenetic β‐diversity is lower than the taxonomic β‐diversity, this would indicate that the communities are mainly composed of closely related species, and if there is no significant difference between phylogenetic and taxonomic β‐diversity, the species turnover is considered independent of phylogeny (Graham et al., 2009).

To assess the effects of environmental and spatial variables on taxonomic and phylogenetic β‐diversity, we expect that at the regional scale, the stress of environmental filtering should be large enough to facilitate species assemblages that were suitable for local conditions, so that closely related species were not easily filtered out. Therefore, we proposed to test the following two hypotheses: (1) Taxonomic β‐diversity (representing dissimilarity) should be highly related to and higher than phylogenetic β‐diversity and (2) the proportion of phylogenetic β‐diversity explained by environmental and spatial variables should be higher than that of taxonomic β‐diversity. Based on census data from 35 1‐ha forest plots established in eight national nature reserves in Guangxi Zhuang Autonomous Region, we quantified the taxonomic and phylogenetic β‐diversity using the (abundance‐based) Rao's quadratic entropy and the (incidence‐based) Sørensen dissimilarity indices. Then, controlling the sampling effect with an individual‐based null model described in Kraft et al. (2011), we calculated the standard effect size of observed β‐diversity deviation (β‐deviation) and partitioned its variation to assess the relative importance of environmental and spatial variables. We expect that a combination of taxonomic and phylogenetic information can allow us to draw more detailed and novel conclusions associated with the mechanisms underlying the formation and maintenance of biodiversity.

## 2 MATERIALS AND METHODS

### 2.1 Study sites

**FIGURE 1:**
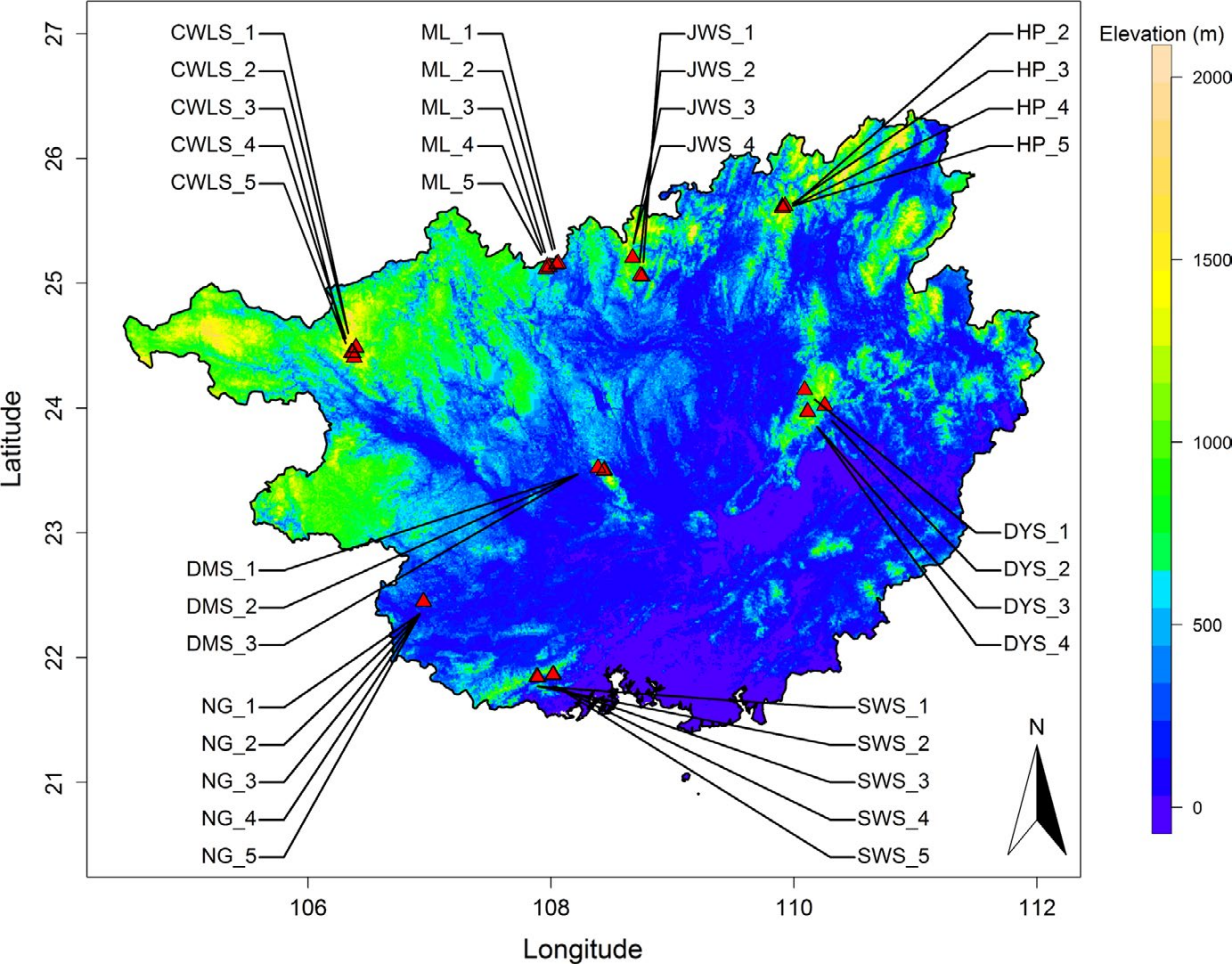
The distribution map of the 35 studied plots in Guangxi. The color background represents the elevation distribution. NG is for Nonggang Nature Reserve, ML for Mulun Nature Reserve, both of which are karst land. The rest sites are nonkarst. SWS is for Shiwandashan Nature Reserve, DMS for Damingshan Nature Reserve, CWLS for Cenwanglaoshan Nature Reserve, JWS for Jiuwanshan Nature Reserve, DYS for Dayaoshan Nature Reserve, and HP for Huaping Nature Reserve

We used the community data of 35 1‐ha tree stem‐mapped plots from eight well‐separated national nature reserves across the Guangxi Zhuang Autonomous Region (hereafter, Guangxi), China (Figure 1). Guangxi is located in the subtropical and northern tropical zones of southern China, and its eastern part is in humid climate and western part in the transitional zone from humid to semi‐humid climate. It is a hilly region with various geomorphological forms, such as mountains or hills with acid and clay soils, plains, and karst landforms. The criteria for the selection of sites for the establishment of plots were based on the regional biodiversity characteristics, choosing across different climatic zones and vegetation types. The plots in each reserve are well separated to represent different floral composition and in the continuous natural forests which are strictly protected. All plots were constructed according to the methodology developed by the Center for Tropical Forest Science (CTFS), Smithsonian Tropical Research Institute. The 35 plots are distributed in eight geographically separated nature reserves, ranging from northern tropical to middle subtropical areas, and each site had 3–5 plots. The census of 35 plots was completed in 2018, recording 160,506 free‐standing individuals with the diameter at breast height (DBH) ≥1 cm and belonging to 975 species, 350 genera, and 102 families (for more details please see Table S1 in Appendix S1).

The surveyed plots spanned a vast spatial range, ranging from 106°21′ to 110°15′E in longitude, from 21°50′ to 25°37′N in latitude, and from 340 m to 1,843 m in elevation (Table S1 in Appendix S1). Additionally, the plots covered various forest types, such as the northern tropical seasonal rainforest (5 plots in Shiwandashan Nature Reserve, hereafter, SWS), northern tropical karst forest (5 plots in Nonggang Nature Reserve, hereafter, NG), subtropical karst forest (5 plots in Mulun Nature Reserve, hereafter, ML), mid‐mountain evergreen and deciduous broad‐leaved mixed forest (5 plots in Cenwanglaoshan Nature Reserve, hereafter, CWLS), and subtropical evergreen broad‐leaved forest (the remaining 15 plots in 4 Reserves).

### 2.2 Environmental and spatial variables

The climatic data were derived from the China ground‐based average dataset of daily meteorological records from 1981 to 2010, recorded by 2,160 base, standard, and ordinary ground‐based meteorological observatories. The data can be downloaded from the China Meteorological Data Service Center (http://data.cma.cn). The dataset included six daily variables: average temperature, average maximum temperature, average minimum temperature, average vapor pressure, average precipitation, and average wind speed.

We also extracted the meteorological data of the 75 observatories distributed in Guangxi. In order to generate more biologically meaningful variables, we used elevation, 19 bioclimatic variables, and the potential evapotranspiration (PET) as the environmental variables, based on the descriptions of bioclimatic variables on the website of Global Climate Data (http://worldclim.org/) and the Penman–Monteith equation recommended by the Food and Agriculture Organization of the United Nations (FAO), respectively. These variables are widely used in species distribution modeling and related ecological modeling (Myers et al., 2013; Xu & Hutchinson, 2011, 2013; Zhang et al., 2020). Finally, using the “mgcv” and “gstat” packages in R, we calculated the values of the 19 bioclimatic variables and PET at each location of 35 plots using a regression kriging approach. However, many intercorrelations were noted among the 19 bioclimatic variables. Therefore, we excluded the variables with a correlation coefficient greater than 0.8 from several other variables using a correlation test. Finally, 10 variables were retained as environmental variables. Furthermore, the spatial eigenfunctions were calculated by the principal coordinates of neighbor matrices (PCNM) using the geographic coordinates of 35 plots (Legendre et al., 2009). The parts of the spatial eigenfunctions with positive eigenvalues were selected to represent the spatial variables (Legendre & Legendre, 2012; Legendre et al., 2009).

### 2.3 Taxonomic and phylogenetic β‐diversity

Based on census data, we generated matrices of taxonomic and phylogenetic β‐diversity, respectively. A phylogeny for 976 species was generated by the “V. PhyloMaker” R‐package (see more details in Jin & Qian, 2019).

We computed the observed β‐diversity for each pairwise plot using the (abundance‐based) standardized Rao's quadratic entropy and the (incidence‐based) Sørensen dissimilarity indices. The algorithm of standardized Rao's quadratic entropy are as follows (Hardy & Senterre, 2007; Rao, 1982; Swenson, 2011):

(1) $$\text{RD}=\sum_{i}\sum_{j}\delta_{\mathrm{ij}}f_{ik_{1}}f_{ik_{2}}$$

(2) $$\text{RH}=\text{RD}‐\frac{\left(\sum_{i}^{S_{k_{1}}}f_{i}\bar{\delta_{k_{1}}}+\sum_{j}^{S_{k_{2}}}f_{j}\bar{\delta_{k_{2}}}\right)}{2}$$

where RD is Rao's quadratic entropy, $\delta_{\mathrm{ij}}$ is the difference between species *i* and species *j* (for generating taxonomic β‐diversity, $\delta_{\mathrm{ij}}$ is 1 when i≠j, $\delta_{\mathrm{ij}}$ is 0 when i=j; for generating phylogenetic β‐diversity, $\delta_{\mathrm{ij}}$ is the pairwise phylogenetic distance between species *i* and species *j*), $f_{ik_{1}}$ is the relative abundance of species *i* in community *k*_1_, $f_{ik_{2}}$ is the relative abundance of species *j* in community *k*_2_, RH is the standardized Rao's quadratic entropy (the taxonomic and phylogenetic β‐diversity are both generated by Equations (1) and (2), hereinafter, RH for taxonomic β‐diversity, PhyloRH for phylogenetic β‐diversity), $S_{k_{1}}$ and $S_{k_{2}}$ represent the richness of community *k*_1_ and community *k*_2_, respectively, $f_{i}$ and $f_{j}$ represent the relative abundance of the *i*‐th species and *j*‐th species, respectively, and $\delta_{k_{1}}$ and $\delta_{k_{2}}$ represent the mean difference of species in community *k*_1_ and community *k*_2_, respectively (for phylogenetic β‐diversity, $\delta_{k_{1}}$ and $\delta_{k_{2}}$ are the mean pairwise phylogenetic distance in community *k*_1_ and community *k*_2_, respectively).

The algorithms of the Sørensen dissimilarity indices are as follows (Baselga, 2010; Bryant et al., 2008; Swenson, 2011):

(3) $$\text{SOR}=1‐\frac{2S_{k_{1}k_{2}}}{\left(S_{k_{1}}+S_{k_{2}}\right)}$$

(4) $$\text{PhyloSOR}=1‐\frac{2{\text{BL}}_{k_{1}k_{2}}}{\left({\text{BL}}_{k_{1}}+{\text{BL}}_{k_{2}}\right)}$$

where SOR is the taxonomic Sørensen dissimilarity index; $S_{k_{1}}$ and $S_{k_{2}}$ represent the number of species in community *k*_1_ and community *k*_2_, respectively; $S_{k_{1}k_{2}}$ is the number of species shared between community *k*_1_ and community *k*_2_; PhyloSOR is the phylogenetic Sørensen dissimilarity index; ${\text{BL}}_{k_{1}k_{2}}$ is the total length of branches shared between community *k*_1_ and community *k*_2_; and ${\text{BL}}_{k_{1}}$ and ${\text{BL}}_{k_{2}}$ represent the total length of branches in community *k*_1_ and community *k*_2_, respectively.

Pearson's correlation analysis was applied to test the correlation between taxonomic and phylogenetic β‐deviation. Considering the potential differences in community assembly between nonkarst and karst forests, we divided the pairwise plots into three classes (NN: pairwise nonkarst forest plots; NK: pairwise nonkarst and karst forest plots; and KK: pairwise karst forest plots) to test whether there were significant differences among them.

### 2.4 Null model and β‐deviation

Based on the approach devised by Kraft et al. (2011), we calculated the standard effect size of the observed β‐diversity deviation (β‐deviation). First, we defined the regional species pool as the total species richness and the total abundance of each species in all 35 plots (Myers et al., 2013). With the precondition of preserving the total abundance of each species in the regional species pool and the number of individuals in each plot, for each pairwise plot, all individuals in the regional species pool were shuffled randomly. This step was repeated 999 times to sample the focal pairwise plots to calculate the mean value and standard deviation of expected β‐diversity using RH, PhyloRH, SOR, and PhyloSOR. Next, we obtained the β‐deviation by dividing the difference of the observed β‐diversity from the expected β‐diversity by the standard deviation of the expected β‐diversity for each pairwise plot as follows:

(5) $$\beta_{\text{dev}}=\frac{\left(\beta_{\text{obs}}‐{\bar{\beta }}_{\text{ran}}\right)}{SD(\beta_{\text{ran}})}$$

where $\beta_{\text{dev}}$ is the β‐deviation, $\beta_{\text{obs}}$ is the observed β‐diversity, and ${\bar{\beta }}_{\text{ran}}$ and $SD(\beta_{\text{ran}})$ represent the expected value and the standard deviation of β‐diversity estimated by 999 species assemblages out of random shuffling, respectively. The significance of differences among observed β‐diversity, expected β‐diversity, and β‐deviation in different classes were tested using the Wilcoxon rank sum test (Myers et al., 2013).

### 2.5 Variation partitioning

We partitioned the variation in taxonomic and phylogenetic β‐diversity and β‐deviation using distance‐based redundancy analysis (dbRDA, Legendre et al., 2009). We constructed a “full model” using the 10 retained environmental variables and the spatial variables approbated by PCNM as predictors and tested the significance of the full model. Using the “ordistep” function in the “vegan” package, the performance of the model was automatically adjusted and optimized step‐by‐step using the forward model selection. According to the final model, the variation in β‐diversity and β‐deviation was partitioned by variables remaining after selection into four parts: (a) purely explained by environmental variables, (b) jointly explained by environmental variables and spatial variables, (c) purely explained by spatial variables, and (d) an unexplained part. As similar results were obtained using Rao's quadratic entropy and Sørensen dissimilarity, we focused on the results of Rao's quadratic entropy in the results section below, while the results of the Sørensen dissimilarity indices are presented in Appendix S2. All analyses were carried out in R (version 3.6.3, R Core Team, 2020).

## 3 RESULTS

**FIGURE 2:**
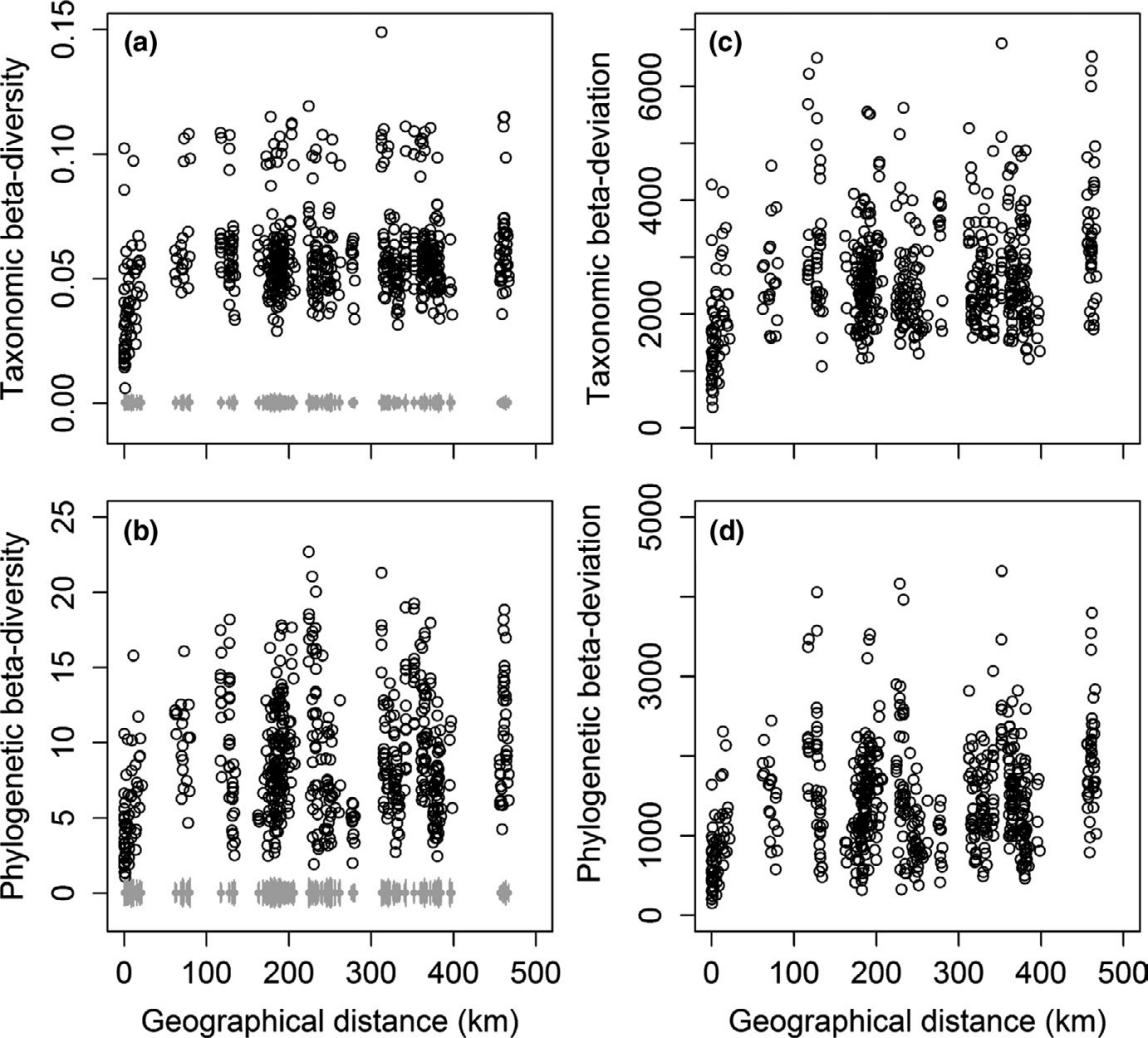
Relationships of taxonomic (a and c) and phylogenetic (b and d) β‐diversity and β‐deviation with geographical distance among 595 pairwise plots. Left panels: taxonomic and phylogenetic observed (black circles) and expected (gray dots with error bars representing 100 standard deviation) β‐diversity with geographic distance are shown in a and b, respectively. Right panels: taxonomic and phylogenetic β‐deviation (black circles) with geographic distance are shown in c and d, respectively

The mean values of the taxonomic and phylogenetic β‐diversity of 595 pairwise plots were 0.06 and 8.87 and ranged from 0.01 to 0.15 and from 1.08 to 22.71, respectively (Figure 2). The mean values of taxonomic and phylogenetic β‐deviation were 2,640.69 and 1,436.03, with variations ranging from 366.81 to 6,756.28 and from 154.74 to 4,326.02, respectively. The taxonomic β‐deviation was highly associated with the phylogenetic β‐deviation (*r* = .74, *p* < .001). Furthermore, the taxonomic and phylogenetic β‐diversities were significantly higher than their own expected β‐diversity and increased with increasing geographical distance (slope_RH. obs_ = 0.04, *p* < .05; slope_PhyloRH. obs_ = 0.88, *p* < .05). Over 98% of all pairwise plots revealed that the taxonomic β‐deviation was on average 107% higher than the corresponding phylogenetic β‐deviation. The higher phylogenetic β‐deviation occurred in seven pairwise plots of karst versus nonkarst forest plots than other pairwise plots.

**FIGURE 3:**
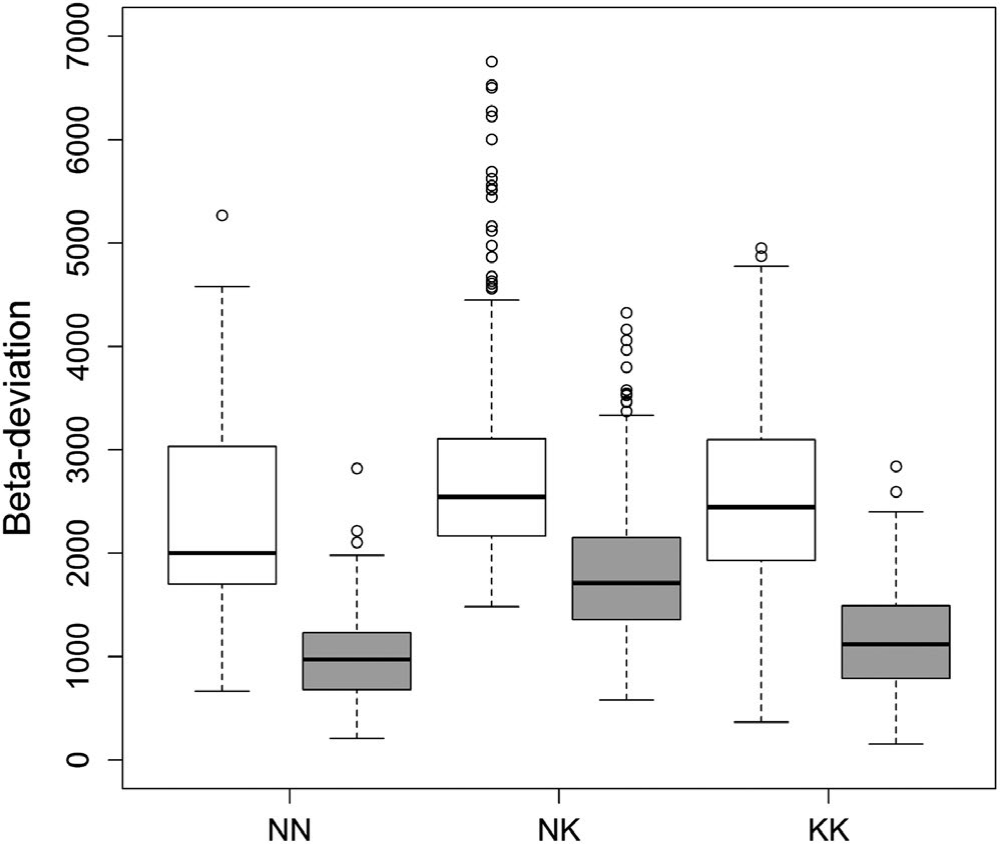
Boxplots of taxonomic (white boxes) and phylogenetic (gray boxes) β‐deviation among three classes. KK, pairwise karst and karst forest plots; NK, pairwise nonkarst and karst forest plots; NN, pairwise nonkarst and nonkarst forest plots

We divided the pairwise plots into three classes. The results revealed that the taxonomic and phylogenetic β‐deviations in the NK pairs were significantly higher than those in the other two classes (*p* < .01; Figure 3 and Figure S4). The results of SOR and PhyloSOR were similar to those of RH and PhyloRH, respectively, but the deviation evaluated by SOR and PhyloSOR varied in a smaller range (Figure S4).

**FIGURE 4:**
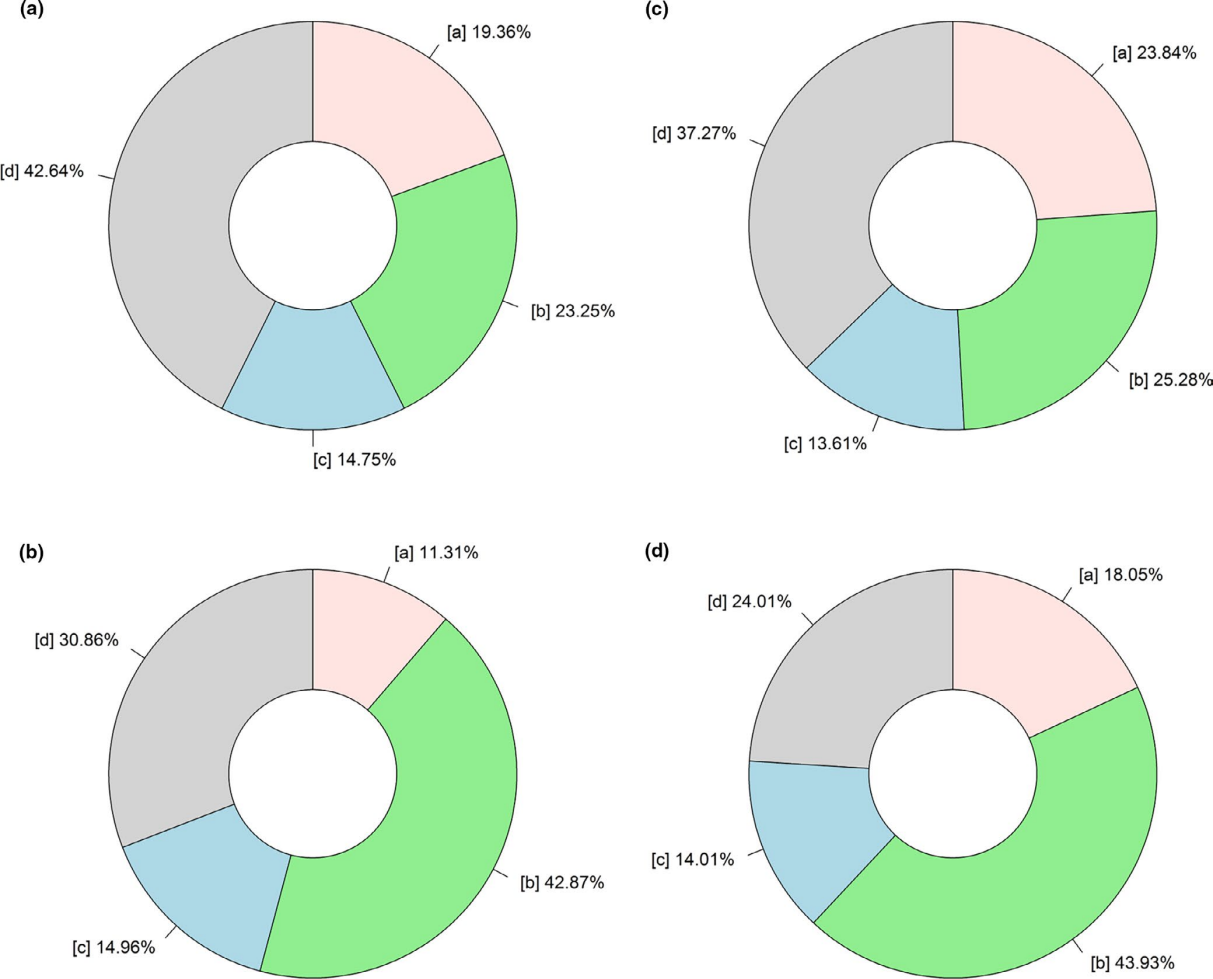
Proportions of variation in taxonomic β‐diversity (a), phylogenetic β‐diversity (b), taxonomic β‐deviation (c), and phylogenetic β‐deviation (d) explained by environmental and spatial variables. [a] variation explained solely by environmental variables, [b] variation explained jointly by environmental and spatial variables, [c] variation explained solely by spatial variables, and [d] unexplained variation

Environmental and spatial variables differed in explaining the variation in taxonomic and phylogenetic β‐deviation (Figure 4 and Figure S5). First, the joint effect of environmental and spatial variables predominantly explained the variation in taxonomic and phylogenetic β‐deviation, but the effect of environmental variables on phylogenetic β‐deviation was lower than that on taxonomic β‐deviation. Second, after controlling for the sampling effect, the total explained proportion of β‐diversity improved. Third, the total explained proportion of taxonomic β‐diversity was lower than that of phylogenetic β‐diversity.

## 4 DISCUSSION

### 4.1 Beta diversity and beta deviation

We evaluated the taxonomic and phylogenetic β‐diversity and analyzed the effects of environmental and spatial factors on the diversity variation using 35 1‐ha natural forest plots across eight nature reserves in Guangxi. Our results showed that the observed taxonomic and phylogenetic β‐diversities were significantly higher than their corresponding expected β‐diversity, suggesting a strong impact of community assembly mechanisms on diversity patterns. Furthermore, taxonomic β‐deviation was highly related to phylogenetic β‐deviation (*R* = .74, *p* < .001) and significantly higher than phylogenetic β‐deviation, being on average 107% higher for the abundance‐based index and 62.14% higher for the incidence‐based index. These results suggested that the community assembly was created by the species with the same or similar phylogenetic components (i.e., the same or similar genera and families) at a regional scale, and thus, the species turnover between pairwise plots was mainly the turnover of closely related species from the same or similar clades (Graham et al., 2009; Qian, Swenson, et al., 2013). A portion of the variation in phylogenetic β‐diversity could be explained by the variation in taxonomic β‐diversity and vice versa (Graham et al., 2009; Qian, Swenson, et al., 2013).

Previous studies have revealed a negative correlation between β‐diversity (similarity) and the dispersal capacity of plant seeds (Qian, 2009; Qian, Chen, et al., 2013). Species with larger propagules were more strongly affected by dispersal limitation than species with smaller propagules, probably resulting in a low dissimilarity in species composition between communities in a close location (Qian & Guo, 2010; Ricklefs et al., 2008). In our study, the increase in geographical distance resulted in increases in the dissimilarity of species composition between plots. Most of genera that recorded in the census data of 35 plots were distributed around the focal plot. However, only 13 genera, including *Cinnamomum*, *Lithocarpus*, *Litsea*, and *Schefflera*, which have relatively small seed sizes, were distributed in all eight nature reserves (Table S3).

We divided the 595 pairwise plots into three classes according to the features of the pairwise plots. The results showed that higher taxonomic and phylogenetic β‐deviation always occurred in the pairwise karst and nonkarst forest plots (Figure 3). These phenomena also occurred when using incidence‐based indices (Figure S4). This suggests that the species composition of karst forests differed from that of other forests compared, with predominantly distantly related clades in the two forest types. In the karst region, the unique geological characteristics and hydrological structure result in unique habitat conditions, such as the slow formation rate of soil, alkaline soil, low water availability, low content of available soil nutrients, and Ca and Mg toxicity to plants (Ferlan et al., 2011; Geekiyanage et al., 2019; Sweeting, 1995). Such harsh conditions require specific biological adaptations of plants. Many species found in the biogeographic region cannot survive and grow in karst landform. The unique habitat conditions result in a unique species composition in the local forests, which differs from that of nonkarst forests in close localities (Zhu et al., 1998, 2003). Compared with climatic factors, complex topographical features and highly heterogeneous and harsh local habitat conditions are likely to be more important drivers of the uniqueness of karst forest communities.

### 4.2 The effect of environmental and spatial variables on beta deviation

The environmental and spatial variables explained the most variation in taxonomic and phylogenetic β‐deviation (Figure 4). This may be due to the large size of our plots. Scale dependence plays a very important role in diversity research (Mykrӓ et al., 2007). The integrality of sampling and species pool increases with increasing scales, which results in more integrated information on community assembly. The size of sampling unit and regional species pool of some studies are inappropriately defined (Tuomisto & Ruokolainen, 2012), thereby resulting in the absence of a large number of rare species in random sampling, and the miscalculation of species’ relative dominance in the community. This can lead to a large underestimation of the effect of environmental or spatial variables on biodiversity (Barton et al., 2013; Xing & He, 2019). The effects of community assembly mechanisms differ at different scales (Xing & He, 2019). Many previous studies have confirmed that species interactions, local habitat heterogeneity, and demographic stochasticity are usually prominent drivers at fine scales (smaller than 0.5‐ha), while regional environmental filtering and dispersal limitation are more important at broader scales (greater than 0.5‐ha) (Chang et al., 2013; Shipley et al., 2012). As the scale increases, the effect of environmental filtering becomes greater (Gilbert & Lechowicz, 2004; Jones et al., 2006). This may explain the high contribution of environmental variables to taxonomic and phylogenetic β‐deviation in our study.

The relative contributions of deterministic and stochastic processes for community assembly have long been debated (Anderson et al., 2011). Some researchers theorize that low‐diversity forests (e.g., temperate forests) reflect strong environmental correlations with the diversity, indicating the important role of niche‐based processes, while high‐diversity forests (e.g., tropical forests) reflect strong spatial correlations with the diversity, indicating the important role of dispersal limitation (Hubbell, 2001; Myers et al., 2013). Our findings do not support this general hypothesis. Some parallel results have been obtained in other studies. For instance, Legendre et al. (2009) found that forest diversity was equally influenced by environmental variables and neutral processes in a 24‐ha plot in the Gutianshan National Nature Reserve in East China. Luo et al.(2019) reported that the relative effects of deterministic processes were more prominent on woody species assemblages at the community scale than on herbaceous species at the neighborhood scale in 15 0.1‐ha plots on Yulong Mountain in Southwest China. Therefore, in subtropical forests that support high woody plant diversity, environmental variables could be better predictors of taxonomic and phylogenetic β‐diversity than spatial variables.

The high contribution of the joint effect of environmental and spatial variables on the β‐diversity deviations of the present study also suggests that environmental filtering is an important process. The joint effect can reflect the spatially structured component of measured and unmeasured environmental factors, and other unknown processes (Smith & Lundholm, 2010). Environmental conditions are often spatially autocorrelated, and they generally have their spatial structures. Thus, spatial factors and environmental variables (including the unmeasured environmental variables in this study) may be generally covariant (Legendre, 1993). In this study, a nested design has been applied for the distribution of the plots. The environmental factors of the neighboring plots in the same and close locations may have relatively higher autocorrelations, which probably contributes to the large joint effect of environmental and spatial variables on the β‐diversity deviations of the present study.

Since the subtropical region of China was not directly affected by the continental ice sheet during the Pleistocene Glaciations, the biodiversity and biomes in the subtropical and northern tropical areas of China are very rich owing to the refuges that allowed many species to survive (Manchester et al., 2009; Qiu et al., 2011). Guangxi spans across the northern tropics, southern subtropics, and middle subtropics. The eastern region of Guangxi is a typical humid zone in southern China, while the western region is the transitional zone from the humid to the semi‐humid climate. The historical frequent climate fluctuations and contemporary climate variations have become the main force driving the speciation, geographical distribution, and genetic structure of existing plant species (Avise, 2000; Gao et al., 2012; Hewitt, 2000; Yu et al., 2017; Zou et al., 2013). Furthermore, Guangxi is a hilly region, with a number of mountains in northern, central and southern areas, and karst land forms in western and northern areas. Such a mountainous topographical structure may have resulted in significant geographic isolation, limiting the migration, dispersal and assemblage of many species distributed in present forests (Sakaguchi et al., 2012; Wang et al., 2015).

In fact, some potential factors that affect diversity were not considered in this study. For instance, the lack of the local habitat variables probably results in an underestimation of deterministic processes (Chang et al., 2013). The legacy effect of disturbance events that we did not take into account may also result in an significant decrease in biodiversity within the community and a change in the relative representation of species in mature forests (Rendón‐Carmona et al., 2009; Zhu, 2017), even a influence on the genetic diversity of the logged species (Lowe et al., 2005). Moreover, the statistical sensitivity of complex spatial model to spatial autocorrelation may disturb the variation partitioning (Borcard & Legendre, 2002; Legendre, 1993; Smith & Lundholm, 2010).

### 4.3 Variable selection and null model

In this study, after the forward model selection we retained four to six environmental variables, which were the factors describing the temperature and precipitation in the coldest and warmest months and growing periods (Table 1). A possible reason was that the factors of water and heat influenced the life history of woody plants, thus affecting the diversity of the community. PET had a significant correlation with taxonomic and phylogenetic β‐deviation (*R*_taxonomic_ = .450, *p* < .001; *R*_phylogenetic_ = .487, *p* < .001) and was retained, showing an important role in explaining the variation in β‐diversity. O'Brien (1993) pointed out that in southern Africa, PET was more indicative of the effect of heat on local plant diversity than temperature. PET, calculated by the Penman–Monteith equation, takes into account the theory of energy balance and water vapor diffusion, reflecting the combined effects of climate factors, thus having a positive effect on the formation and variation in the β‐diversity pattern explained by the water‐energy dynamic hypothesis (van Bavel, 1966).

**TABLE 1:** The environmental and spatial variables used in the models for partitioning the variation in taxonomic and phylogenetic β‐deviation

	Selected environmental variables	Selected spatial variables
Full model	Bio2, Bio4, Bio5, Bio7, Bio10, Bio11, Bio12, Bio13, Bio14, Bio15, Bio18, PET, Elevation	PCNM1, PCNM2, PCNM3, PCNM4, PCNM5, PCNM6, PCNM7, PCNM8, PCNM9, PCNM10, PCNM11, PCNM12, PCNM13, PCNM14, PCNM15, PCNM16, PCNM17
Model selection		
Taxonomic β‐deviation	Bio2, Bio5, Bio10, Bio13, Bio18, PET	PCNM1, PCNM2, PCNM3, PCNM4, PCNM5, PCNM9
Phylogenetic β‐deviation	Bio11, Bio12, Bio18, PET	PCNM1, PCNM2, PCNM3, PCNM4, PCNM5, PCNM7, PCNM9

Note

The variables used in the full model and model selection were those obtained after the exclusion of highly correlated variables and those obtained after excluding the highly correlated variables and forward model selection, respectively.

The null model of Kraft et al. (2011) controls the sampling effect caused by the abundance distribution of species in the regional species pool, making the β‐deviation comparable. Our results were similar to those of Myers et al. (2013) in that the explained proportion of variation in taxonomic and phylogenetic β‐diversity significantly increased after controlling for the sampling effect, especially the portion explained by environmental variables. This result suggests that the importance of stochastic processes was overestimated in the observed β‐diversity because of the sampling effect, especially the relative difference between the portion explained by deterministic and stochastic processes.

## 5 CONCLUSIONS

Our study highlights the importance of understanding community assembly mechanisms from the perspective of a combination of taxonomic and phylogenetic β‐diversity. In our study system, the species turnover between pairwise plots was mainly the turnover of closely related species. However, the significant difference in taxonomic and phylogenetic β‐deviation between pairwise karst and nonkarst forest plots and other pairwise plots suggested a huge dissimilarity in the composition of species from distantly related clades in karst communities to neighboring nonkarst forest communities. The current regional taxonomic and phylogenetic diversity patterns probably resulted from the impact of unique climate and geomorphic characteristics. Thus, deterministic processes were responsible for the diversity observed in the plots.

## CONFLICT OF INTEREST

The authors declare that they have no conflict of interest.

## AUTHOR CONTRIBUTIONS

**Wei Shi:** Conceptualization (equal); Formal analysis (lead); Investigation (equal); Methodology (equal); Project administration (supporting); Resources (equal); Validation (lead); Visualization (lead); Writing‐original draft (lead); Writing‐review & editing (supporting). **Yong‐Qiang Wang:** Formal analysis (supporting); Investigation (equal); Methodology (equal); Validation (supporting); Visualization (supporting). **Wu‐Sheng Xiang:** Data curation (equal); Investigation (equal); Methodology (supporting); Project administration (supporting); Resources (equal). **Xian‐Kun Li:** Data curation (equal); Investigation (equal); Methodology (supporting); Project administration (supporting); Resources (equal). **Kun‐Fang Cao:** Conceptualization (equal); Data curation (equal); Methodology (equal); Project administration (lead); Resources (equal); Supervision (lead); Writing‐review & editing (lead).

## Supporting information

Appendix S1

Appendix S2

## Data Availability

The taxonomic and phylogenetic data of all pairwise plots and the environmental variables of 35 plots will be archived in Dryad Digital Repository (https://doi.org/10.5061/dryad.05qfttf20). Until the manuscript is published, please use the following link: https://datadryad.org/stash/share/i8HkM8VgzO6vwStH4‐XDgFn‐hX7R08tl6QRC9yaPbbk.
