# Supplementary material for: Environmental filtering and dispersal limitation jointly shaped the taxonomic and phylogenetic beta diversity of natural forests in southern China
Source: Ecol Evol. 2021 May 26;11(13):8783–94. doi: 10.1002/ece3.7711 (PMC8258218; doi:10.1002/ece3.7711)
Supplement: Supplementary file 1 — Appendix S1 [file ECE3-11-8783-s001.docx]

**Table S1** Details of the 35 studied plots.

| Plots | Attributions | Longitude | Latitude | Elevation | Families | Genera | Species richness | The number of individuals |
| --- | --- | --- | --- | --- | --- | --- | --- | --- |
| CWLS_1 | Cenwanglaoshan National  Nature Reserve | 106.3944 | 24.4885 | 1843 | 26 | 55 | 118 | 5095 |
| CWLS_2 | Cenwanglaoshan National  Nature Reserve | 106.3948 | 24.4836 | 1745 | 33 | 54 | 117 | 3185 |
| CWLS_3 | Cenwanglaoshan National  Nature Reserve | 106.3579 | 24.4450 | 1366 | 46 | 89 | 164 | 4063 |
| CWLS_4 | Cenwanglaoshan National  Nature Reserve | 106.3707 | 24.4420 | 1492 | 39 | 74 | 139 | 4716 |
| CWLS_5 | Cenwanglaoshan National  Nature Reserve | 106.3793 | 24.4054 | 1546 | 40 | 75 | 135 | 2254 |
| DMS_1 | Damingshan National  Nature Reserve | 108.3865 | 23.5195 | 1147 | 38 | 68 | 97 | 5091 |
| DMS_2 | Damingshan National  Nature Reserve | 108.4336 | 23.4950 | 1223 | 38 | 59 | 83 | 6373 |
| DMS_3 | Damingshan National  Nature Reserve | 108.4410 | 23.5032 | 1225 | 30 | 49 | 71 | 5674 |
| DYS_1 | Dayaoshan National  Nature Reserve | 110.0875 | 24.1453 | 655 | 46 | 77 | 117 | 3347 |
| DYS_2 | Dayaoshan National  Nature Reserve | 110.2525 | 24.0208 | 526 | 47 | 73 | 122 | 3702 |
| DYS_3 | Dayaoshan National  Nature Reserve | 110.1108 | 23.9723 | 1321 | 32 | 53 | 92 | 2884 |
| DYS_4 | Dayaoshan National  Nature Reserve | 110.1134 | 23.9690 | 1232 | 32 | 59 | 85 | 3985 |
| HP_2 | Huaping National  Nature Reserve | 109.9255 | 25.6191 | 950 | 40 | 80 | 117 | 3478 |
| HP_3 | Huaping National  Nature Reserve | 109.9037 | 25.6150 | 839 | 37 | 69 | 191 | 5117 |
| HP_4 | Huaping National  Nature Reserve | 109.9035 | 25.6029 | 760 | 44 | 79 | 125 | 5773 |
| HP_5 | Huaping National  Nature Reserve | 109.9058 | 25.6018 | 849 | 37 | 70 | 106 | 5630 |
| JWS_1 | Jiuwanshan National  Nature Reserve | 108.7517 | 25.0545 | 626 | 39 | 66 | 94 | 2671 |
| JWS_2 | Jiuwanshan National  Nature Reserve | 108.7348 | 25.0626 | 661 | 41 | 68 | 98 | 4135 |
| JWS_3 | Jiuwanshan National  Nature Reserve | 108.6718 | 25.2043 | 1264 | 39 | 60 | 93 | 4925 |
| JWS_4 | Jiuwanshan National  Nature Reserve | 108.6719 | 25.2063 | 1275 | 41 | 66 | 101 | 6025 |
| ML_1 | Mulun National  Nature Reserve | 108.0581 | 25.1602 | 368 | 42 | 76 | 96 | 2448 |
| ML_2 | Mulun National  Nature Reserve | 108.0436 | 25.1517 | 414 | 46 | 97 | 127 | 2967 |
| ML_3 | Mulun National  Nature Reserve | 107.9860 | 25.1317 | 527 | 45 | 98 | 133 | 3932 |
| ML_4 | Mulun National  Nature Reserve | 107.9694 | 25.1361 | 535 | 48 | 103 | 133 | 3013 |
| ML_5 | Mulun National  Nature Reserve | 107.9585 | 25.1140 | 547 | 38 | 77 | 93 | 2883 |
| NG_1 | Nonggang National  Nature Reserve | 106.9521 | 22.4501 | 190 | 46 | 101 | 124 | 4248 |
| NG_2 | Nonggang National  Nature Reserve | 106.9502 | 22.4504 | 306 | 43 | 84 | 104 | 5508 |
| NG_3 | Nonggang National  Nature Reserve | 106.9492 | 22.4499 | 320 | 43 | 89 | 116 | 4864 |
| NG_4 | Nonggang National  Nature Reserve | 106.9492 | 22.4487 | 317 | 46 | 102 | 129 | 3803 |
| NG_5 | Nonggang National  Nature Reserve | 106.9504 | 22.4492 | 259 | 44 | 94 | 119 | 3692 |
| SWS_1 | Shiwandashan National  Nature Reserve | 107.8912 | 21.8421 | 562 | 56 | 116 | 192 | 7536 |
| SWS_2 | Shiwandashan National  Nature Reserve | 107.8817 | 21.8475 | 655 | 51 | 97 | 158 | 6112 |
| SWS_3 | Shiwandashan National  Nature Reserve | 108.0143 | 21.8625 | 432 | 48 | 97 | 130 | 5910 |
| SWS_4 | Shiwandashan National  Nature Reserve | 108.0208 | 21.8662 | 340 | 54 | 106 | 159 | 7933 |
| SWS_5 | Shiwandashan National  Nature Reserve | 108.0174 | 21.8661 | 470 | 56 | 113 | 171 | 7534 |

**Table S2** Meanings of the 19 bioclimatic variables.

| Variables | Meaning |
| --- | --- |
| Bio1 | Annual mean temperature |
| Bio2 | Mean diurnal range (mean of monthly (max temp - min temp)) |
| Bio3 | Isothermality (Bio2/Bio7) (* 100) |
| Bio4 | Temperature seasonality (standard deviation *100) |
| Bio5 | Max temperature of warmest month |
| Bio6 | Min temperature of coldest month |
| Bio7 | Temperature annual range (Bio5-Bio6) |
| Bio8 | Mean temperature of wettest quarter |
| Bio9 | Mean temperature of driest quarter |
| Bio10 | Mean temperature of warmest quarter |
| Bio11 | Mean temperature of coldest quarter |
| Bio12 | Annual precipitation |
| Bio13 | Precipitation of wettest month |
| Bio14 | Precipitation of driest month |
| Bio15 | Precipitation seasonality (coefficient of variation) |
| Bio16 | Precipitation of wettest quarter |
| Bio17 | Precipitation of driest quarter |
| Bio18 | Precipitation of warmest quarter |
| Bio19 | Precipitation of coldest quarter |
