# Supplementary material for: Environmental filtering and dispersal limitation jointly shaped the taxonomic and phylogenetic beta diversity of natural forests in southern China
Source: Ecol Evol. 2021 May 26;11(13):8783–94. doi: 10.1002/ece3.7711 (PMC8258218; doi:10.1002/ece3.7711)
Supplement: Supplementary file 2 — Appendix S2 [file ECE3-11-8783-s002.docx]

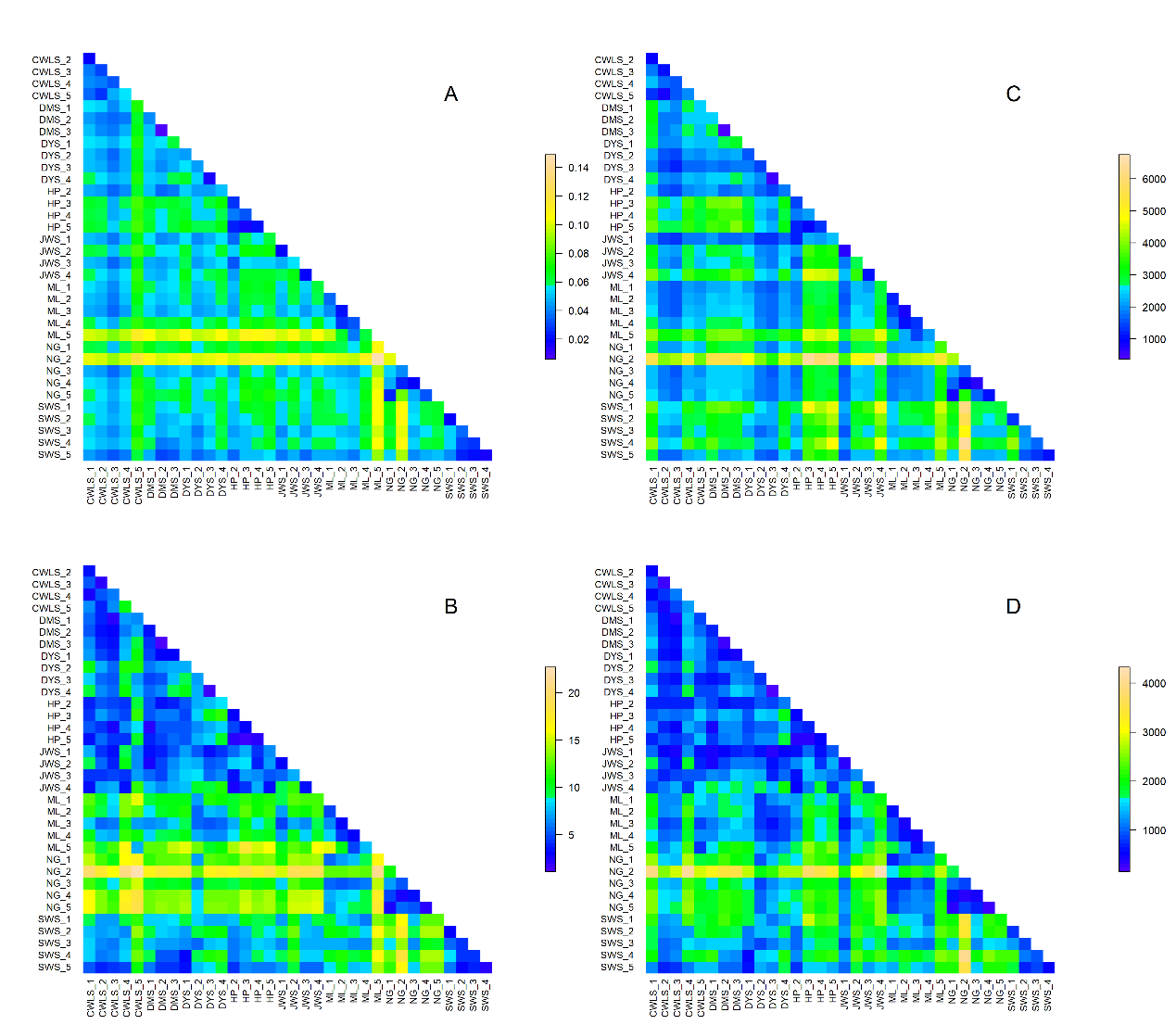


**Figure S1** Metrices of taxonomic β-diversity (A), phylogenetic β-diversity (B), taxonomic β-deviation (C) and phylogenetic β-deviation (D) among 595 pairwise plots evaluated using RH and PhyloRH.


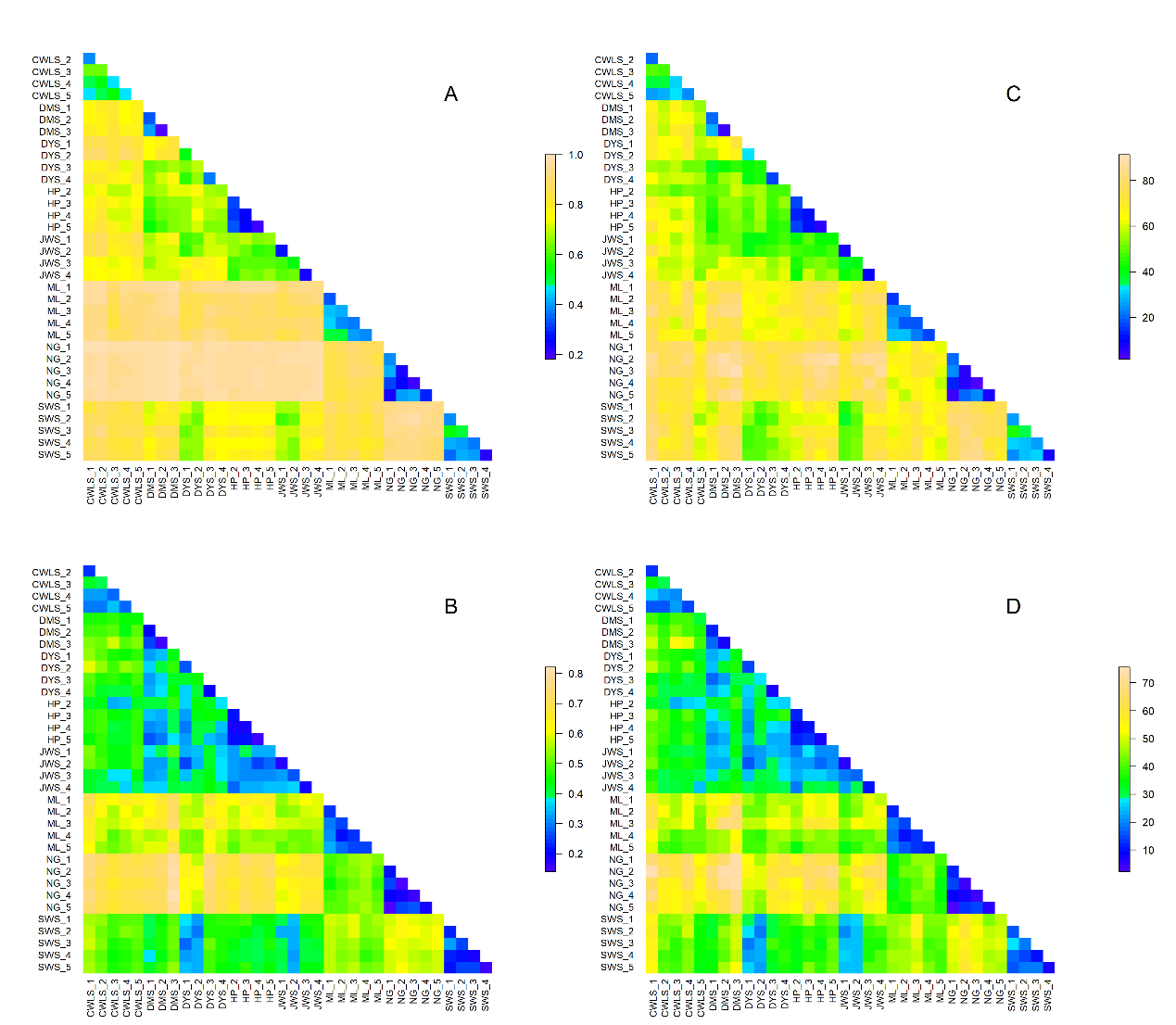


**Figure S2** Metrices of taxonomic β-diversity (A), phylogenetic β-diversity (B), taxonomic β-deviation (C) and phylogenetic β-deviation (D) among 595 pairwise plots evaluated using SOR and PhyloSOR.


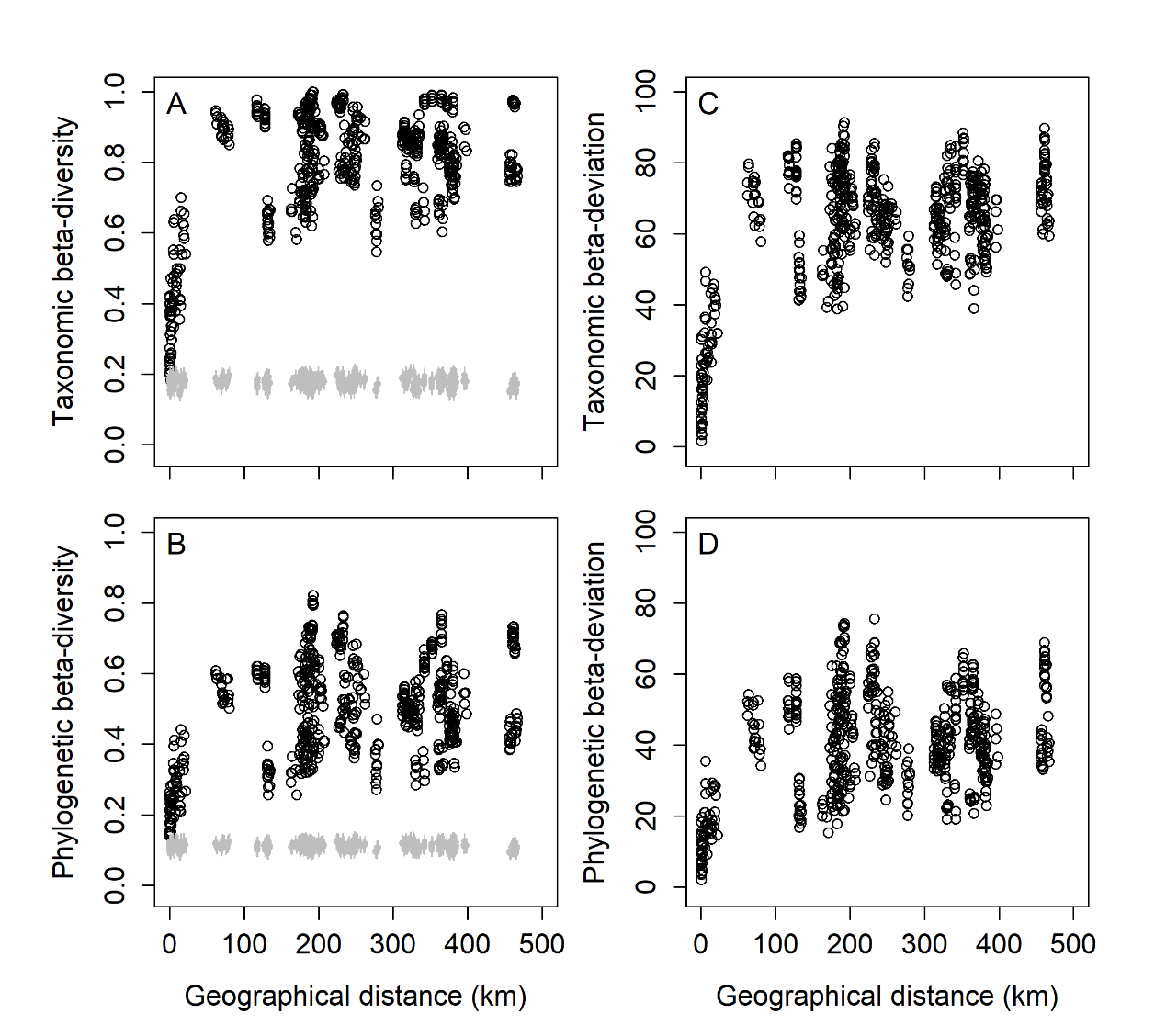


**Figure S3** Relationships of taxonomic (A and C) and phylogenetic (B and D) β-diversity and β-deviation with geographical distance among 595 pairwise plots. Left panels: taxonomic and phylogenetic observed (black circles) and expected (gray dots with error bars representing 2 standard deviation) β-diversity with geographic distance are shown in A and B, respectively. Right panels: taxonomic and phylogenetic β-deviation (black circles) with geographic distance are shown in C and D, respectively. All β-diversities and β-deviations were evaluated using SOR and PhyloSOR.


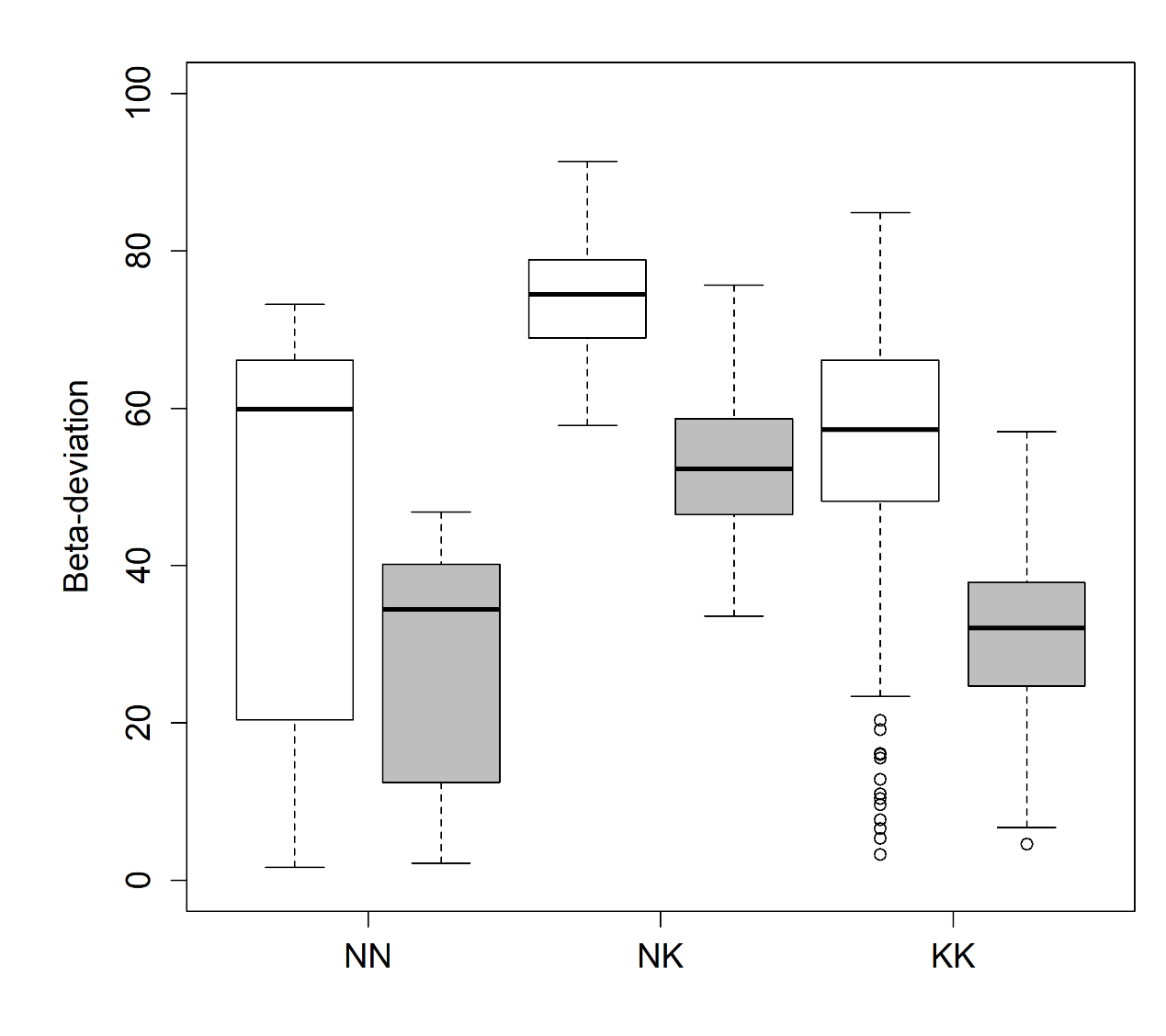


**Figure S4** Boxplots of taxonomic (write boxes with error bars) and phylogenetic (gray boxes with error bars) β-deviation among three classes. NN: pairwise non-karst and non-karst forest plots; NK: pairwise non-karst and karst forest plots; KK: pairwise karst and karst forest plots. All β-diversities and β-deviations were evaluated by SOR and PhyloSOR.


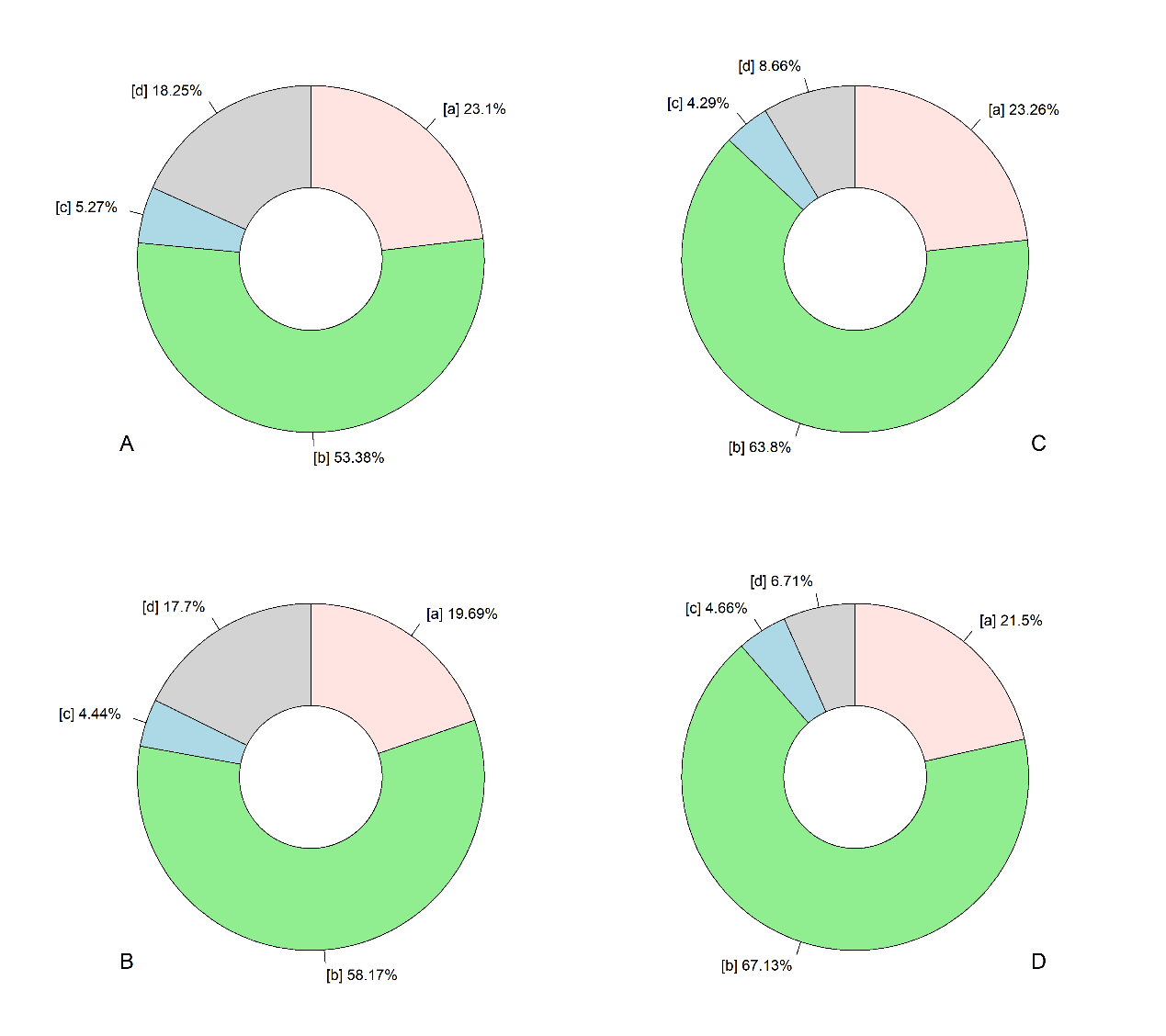


**Figure S5** Proportions of variation in taxonomic β-diversity (A), phylogenetic β-diversity (B), taxonomic β-deviation (C) and phylogenetic β-deviation (D) explained by environmental and spatial variables. [a] variation explained purely by environmental variables, [b] variation explained jointly by environmental and spatial variables, [c] variation explained purely by spatial variables and [d] unexplained variation. All β-diversities and β-deviations were evaluated by SOR and PhyloSOR.

**Table S3** The occurrence of 350 genera distributed among National Nature Reserves.

| Distribution range of genus | Number of genera |
| --- | --- |
| Shared among 8 National Nature Reserves | 13 |
| Shared among 6-7 National Nature Reserves | 46 |
| Shared among 4-5 National Nature Reserves | 46 |
| Shared among 2-3 National Nature Reserves | 104 |
| Occurred in only 1 National Nature Reserve | 141 |

**Table S4** The environmental and spatial variables used in the models for partitioning the variation in taxonomic and phylogenetic β-deviation evaluated using SOR and PhyloSOR. The variables used in the full model were those obtained after the exclusion of high correlative variables. The variables used in model selection were those obtained after excluding the high correlative variables and forward model selection.

|  | Selected environmental variables | Selected spatial variables |
| --- | --- | --- |
| **Full model** | Bio2, Bio4, Bio5, Bio7, Bio10, Bio11, Bio12, Bio13, Bio14, Bio15, Bio18, PET, Elevation | PCNM1, PCNM2, PCNM3, PCNM4, PCNM5, PCNM6, PCNM7, PCNM8, PCNM9, PCNM10, PCNM11, PCNM12, PCNM13, PCNM14, PCNM15, PCNM16, PCNM17 |
| **Model selection** |  |  |
| Taxonomic β-deviation | Bio2, Bio4, Bio5, Bio7, Bio10, Bio11, Bio12, Bio14, Bio18, PET, Elevation | PCNM1, PCNM2, PCNM3, PCNM4, PCNM5, PCNM8, PCNM9 |
| Phylogenetic β-deviation | Bio2, Bio4, Bio5, Bio7, Bio10, Bio12, Bio13, Bio14, PET, Elevation | PCNM1, PCNM2, PCNM3, PCNM4, PCNM5, PCNM7, PCNM9 |

**Table S5** The Pearson correlation coefficients between taxonomic and phylogenetic β-deviation and environmental variables.

| Variables | Rao's quadratic entropy | | Sørensen dissimilarity index | |
| --- | --- | --- | --- | --- |
|  | Taxonomic beta-deviation | Phylogenetic beta-deviation | Taxonomic beta-deviation | Phylogenetic beta-deviation |
| Bio1 | 0.203^***^ | 0.483^***^ | 0.594^***^ | 0.629^***^ |
| Bio2 | 0.111^**^ | 0.134^**^ | 0.328^***^ | 0.227^***^ |
| Bio3 | -0.004 | -0.066 | 0.215^***^ | 0.148^***^ |
| Bio4 | 0.098^*^ | -0.025 | 0.225^***^ | 0.142^***^ |
| Bio5 | 0.067 | 0.298^***^ | 0.527^***^ | 0.606^***^ |
| Bio6 | 0.278^***^ | 0.504^***^ | 0.528^***^ | 0.535^***^ |
| Bio7 | 0.177^***^ | 0.027 | 0.210^***^ | 0.101^*^ |
| Bio8 | 0.133^**^ | 0.394^***^ | 0.572^***^ | 0.621^***^ |
| Bio9 | 0.259^***^ | 0.495^***^ | 0.535^***^ | 0.538^***^ |
| Bio10 | 0.089^*^ | 0.317^***^ | 0.530^***^ | 0.581^***^ |
| Bio11 | 0.284^***^ | 0.493^***^ | 0.515^***^ | 0.510^***^ |
| Bio12 | 0.212^***^ | 0.240^***^ | 0.456^***^ | 0.399^***^ |
| Bio13 | 0.308^***^ | 0.440^***^ | 0.421^***^ | 0.461^***^ |
| Bio14 | 0.206^***^ | 0.102^*^ | 0.347^***^ | 0.263^***^ |
| Bio15 | 0.065 | 0.062 | 0.280^***^ | 0.173^***^ |
| Bio16 | 0.281^***^ | 0.511^***^ | 0.513^***^ | 0.574^***^ |
| Bio17 | 0.159^***^ | 0.068 | 0.342^***^ | 0.276^***^ |
| Bio18 | 0.206^***^ | 0.247^***^ | 0.284^***^ | 0.150^***^ |
| Bio19 | 0.156^***^ | 0.076 | 0.343^***^ | 0.269^***^ |
| PET | 0.254^***^ | 0.450^***^ | 0.501^***^ | 0.487^***^ |
| Elevation | 0.066 | 0.259^***^ | 0.480^***^ | 0.523^***^ |

*** *P* < 0.001; ** *P* < 0.01; * *P* < 0.05
